# CD43 signals induce Type One lineage commitment of human CD4^+ ^T cells

**DOI:** 10.1186/1471-2172-8-30

**Published:** 2007-11-23

**Authors:** Oscar Ramírez-Pliego, Diana L Escobar-Zárate, Gemma M Rivera-Martínez, Mayte G Cervantes-Badillo, Fernando R Esquivel-Guadarrama, Gabriela Rosas-Salgado, Yvonne Rosenstein, M Angélica Santana

**Affiliations:** 1 Facultad de Ciencias, Universidad Autónoma del Estado de Morelos, Av. Universidad 1001, Col. Chamilpa, Cuernavaca, 62210, Mexico; 2 Facultad de Medicina, Universidad Autónoma del Estado Morelos, Av. Universidad 1001, Col. Chamilpa, Cuernavaca, 62210, Mexico; 3 Instituto de Biotecnología Universidad Nacional Autónoma de México, Av. Universidad 2001, Col. Cuernavaca 62210, Mexico

## Abstract

**Background:**

The activation and effector phenotype of T cells depend on the strength of the interaction of the TcR with its cognate antigen and additional signals provided by cytokines and by co-receptors. Lymphocytes sense both the presence of an antigen and also clues from antigen-presenting cells, which dictate the requisite response. CD43 is one of the most abundant molecules on the surface of T cells; it mediates its own signalling events and cooperates with those mediated by the T cell receptor in T cell priming. We have examined the role of CD43 signals on the effector phenotype of adult CD4^+ ^and CD8^+ ^human T cells, both alone and in the presence of signals from the TcR.

**Results:**

CD43 signals direct the expression of IFNγ in human T cells. In freshly isolated CD4^+ ^T cells, CD43 signals potentiated expression of the IFNγ gene induced by TcR activation; this was not seen in CD8^+ ^T cells. In effector cells, CD43 signals alone induced the expression of the IFNγ gene in CD4^+ ^T cells and to a lesser extent in CD8^+ ^cells. The combined signals from CD43 and the TcR increased the transcription of the T-bet gene in CD4^+ ^T cells and inhibited the transcription of the GATA-3 gene in both populations of T cells, thus predisposing CD4^+ ^T cells to commitment to the T1 lineage. In support of this, CD43 signals induced a transient membrane expression of the high-affinity chains of the receptors for IL-12 and IFNγ in CD4^+ ^T cells. CD43 and TcR signals also cooperated with those of IL-12 in the induction of IFNγ expression. Moreover, CD43 signals induced the co-clustering of IFNγR and the TcR and cooperated with TcR and IL-12 signals, triggering a co-capping of both receptors in CD4^+ ^populations, a phenomenon that has been associated with a T1 commitment.

**Conclusion:**

Our results suggest a key role for CD43 signals in the differentiation of human CD4^+ ^T cells into a T1 pattern.

## Background

When T cells encounter antigen-presenting cells (APC) loaded with a peptide that they specifically recognize, they mature to become effector cells [1]. There are three major sub-populations of effector cells. Type One (T1) cells secrete IFNγ, IL-2 and TNFβ and mediate a systemic cellular immune response, through the activation of macrophages and cytotoxic T cells [2,3]. Type Two (T2) cells secrete IL-4, IL-5 and IL-13, and potentiate the isotype switching of immunoglobulins to IgG1 and IgE, promoting neutralizing activity and degranulation of mast cells, thereby inducing a barrier immunity [4]. The Type 17 (T17) cells, recently described, produce IL-17A and F, G-CSF and the chemokines CXCL9, CXCL10 and CXCL11. It promotes life and differentiation of neutrophils and is important in the clearance of extracellular bacteria [5]. Naïve cells can also differentiate into regulatory cells, either TH3 (TGFβ producers), T_R1 _(IL-10 producers) or iT_REG _(IL-10 and TGFβ producers) [6]. Differentiation of cells into T1 or T2 effector cells has been shown mostly to occur in CD4^+ ^and CD8^+ ^T cells, although other immune cells also differentiate into these two patterns [1].

The clone-specific T cell response is provided by signals from the T cell receptor (TcR). Yet additional signals, provided by cytokines and by co-receptors, are also required for the activation and for the determination of the cytokine profile of T cells. Thus, a lymphocyte senses not only the presence of an antigen but also its environment and a particular cellular response will result from the integration of signals delivered by the antigen – specific receptor and the numerous co-receptors and cytokine receptors [7].

The initial signals of differentiation can occur in the absence of cytokines [8]. The stabilization of the differentiated phenotype, however, is thought to depend mostly on cytokines [9]. The cytokines IL-12 and IL-4 play a direct role in the differentiation of lymphocytes into the T1 or T2 patterns, respectively. When activated T cells are cultured in the presence of IL-12 and blocking antibodies against IL-4, they differentiate into the T1 pattern. In the same way, activated cells cultured in the presence of IL-4 and blocking antibodies against IFNγ differentiate into T2 cells [4]. An extensive amount of work has documented the direct involvement of cytokines in the *in vivo *differentiation of T cells into the T1 or T2 patterns [10].

CD43 is a very large and heavily glycosylated molecule, very abundant on the T cell surface [11]. It was originally proposed that its main function was to repulse the interactions between the APC and the T cell, because of its strong negative charge due to the abundance of sialic acid, and extended nature [12]. In addition, during the rearrangement of molecules that accompanies antigen – specific T cell activation, CD43 is excluded from the T-cell – APC contact region, which contains the TcR, as well as other co-receptor molecules [13]. CD43 exclusion from the immunological synapses is an active phenomenon, which gives rise to the formation of a distal complex, probably with signalling activity [14,15]. Even so, the presence of the extracellular domain of CD43 in the contact area between the APC and the T cell does not affect the T cell response [16]. Furthermore, CD43 mediates its own signalling events and cooperates with those mediated by the TcR in T cell priming, as determined in total populations of T cells [17-20].

We evaluated the role of CD43 signals, alone or combined with TcR or IL-12 signals on: a) the expression of IFNγ and IL-4, b) the transcription of T-bet and GATA-3 genes, c) the membrane expression of IL-12R and IFNγR and d) the distribution of IFNγR and the TcR on the surface of human T cells. Our results show that CD43 signals promote T cell commitment to the T1 differentiation pattern in adult T cells, particularly the CD4^+ ^subset.

## Results

### CD43 signals induce IFNγ gene expression

**Figure 1:**
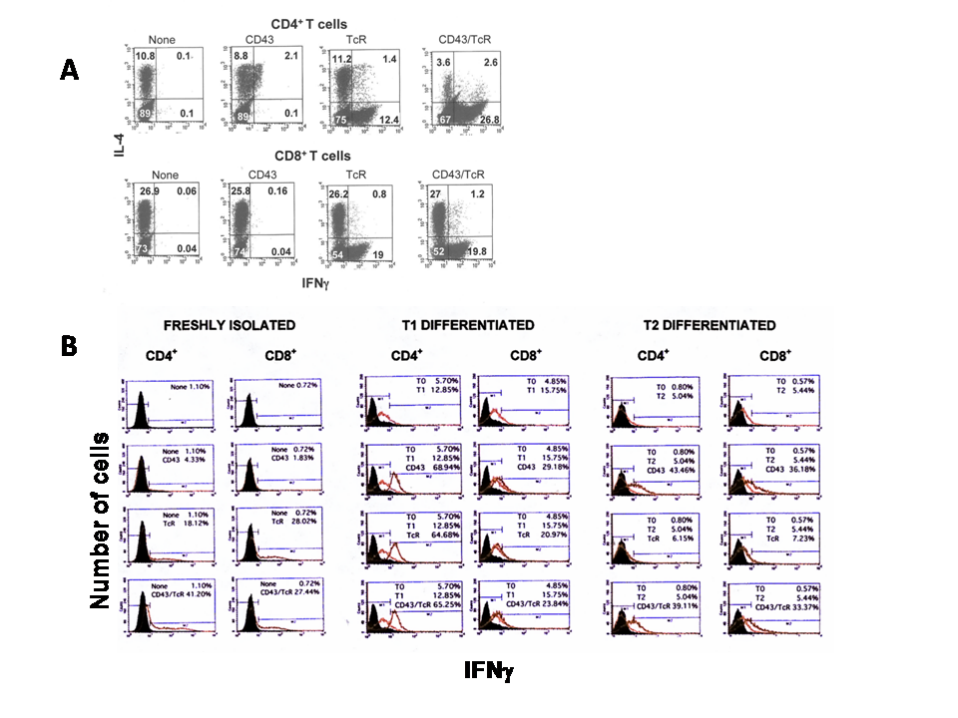
**CD43 and TcR signals induce the expression of IFNγ in CD4^+ ^T cells**. CD4^+ ^or CD8^+ ^T cells were used freshly isolated or after differentiation for one week under T1 or T2 polarizing conditions. Panel A shows the simultaneous analysis of IL-4 and IFNγ expression in freshly isolated cells. In Panel B we show the comparison of IFNγ expression in freshly isolated cells (left panel), and in T1 and T2 differentiated populations. Cells were left non-stimulated or were stimulated for 12 h by antibody ligation of CD3 (TcR), CD43 or the simultaneous ligation of both molecules. Cells were collected and stained with FITC-labelled anti-IFNγ antibody and analyzed by flow cytometry. Representative experiments, out of at least three are shown, using CD4^+ ^or CD8^+ ^T cells from the same donor.

We evaluated the role of CD43 signals in the commitment of human CD4^+ ^and CD8^+ ^T cells into IFNγ producers. In freshly isolated cells, CD43 – ligation induced a modest change in the percentage of IFNγ^+ ^cells in CD4^+ ^but not in CD8^+ ^T cells. Engagement of the TcR led to a substantial increase in the percentage of IFNγ^+ ^cells both in the CD4^+ ^and CD8^+ ^T cell populations. The simultaneous ligation of CD43 and the TcR strongly increased the proportion of IFNγ^+ ^CD4^+ ^T cells, but not that of CD8^+ ^T cells (Figure 1A and 1B, left panel). The CD43 plus TcR induced increase in IFNγ^+ ^CD4^+ ^T cells is accompanied by a simultaneous decrease in IL-4^+ ^cells (Figure 1A).

Next, we evaluated the effect of CD43 signals on cells differentiated for one week under T1 or T2 polarizing conditions (Figure 1B) [21]. In T1 cells, CD43 or the TcR independently were sufficient to induce the expression of IFNγ in CD4^+ ^T cells (68% and 64% over 12.8% in untreated cells), and to a lower extent in CD8^+ ^cells (26.3% and 18.5% over 14% in untreated cells). In the T2 effector cells, CD43, but not the TcR, induced the expression of IFNγ, in both CD4^+ ^and CD8^+ ^cells (43.4% and 38.1% over 5% and 6% in the untreated cells). These results indicate that in CD4^+ ^human T cells, CD43 signals induce a T1 cytokine pattern. In freshly isolated cells, CD43 signals require in addition signals from the TcR but not in either T1 or T2 differentiated cells. Furthermore, in T2 differentiated CD8^+ ^T cells, CD43, but not the TcR, signals induced the expression of IFNγ. A significant increase in IFNγ expression was also observed in response to the joint signals of CD43 and the TcR in freshly isolated cells depleted of CD45RO^+ ^(memory) cells and in human neonatal T cells (data not shown and manuscript in preparation).

**Figure 2:**
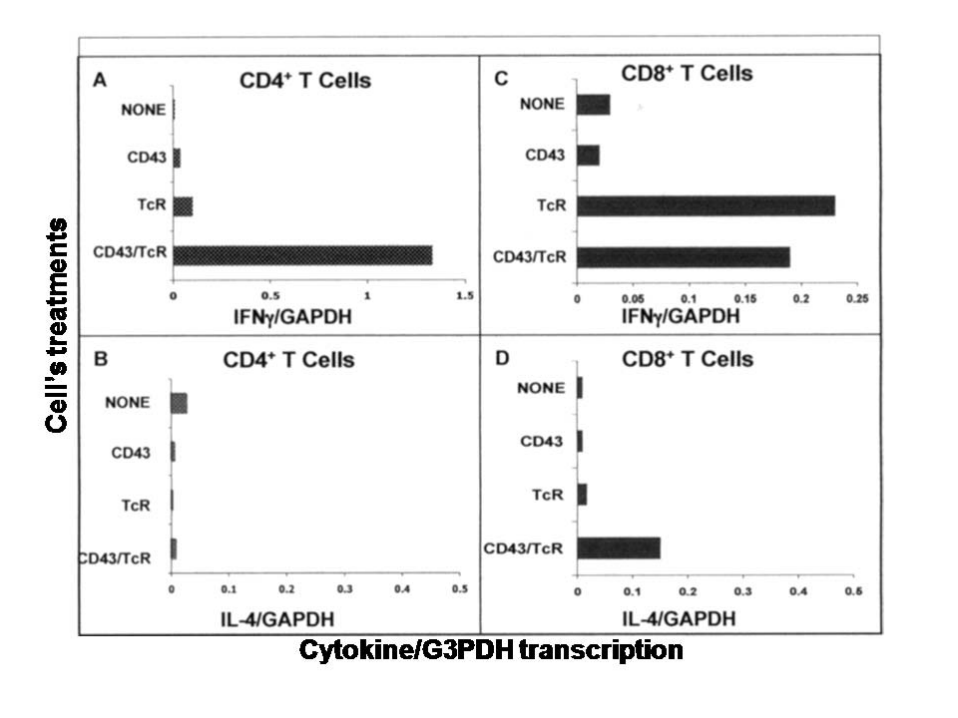
**CD43 and TcR signals induce the transcription of IFNγ in CD4^+ ^and IL-4 in CD8^+ ^T cells**. T cells were treated for 6 h as indicated in Figure 1. Total RNA was extracted from CD4^+ ^(panel A and C) or CD8^+ ^T cells (panel B and D) and subjected to real time RT-PCR analysis for IFNγ (panels A and B) or IL 4 (panels C and D) mRNA contents. A representative of at least 3 independent experiments is shown. Each point is the mean of duplicates which differed by <2%.

In freshly isolated CD4^+ ^T cells, the increase in the percentage of IFNγ^+ ^cells in response to CD43 signals correlated with a decrease of IL-4^+ ^cells (Figure 1A). We thus investigated the effect of CD43 signals in the transcription of the IFNγ and IL-4 genes (Figure 2). In CD4^+ ^T cells, the joint signals of CD43 and the TcR strongly induced IFNγ gene transcription (panel A). Neither CD43 nor the TcR signals alone were sufficient to induce IFNγ gene transcription. Alone or in combination, CD43 and the TcR were inhibitory of basal IL-4 transcription (panel B). In CD8^+ ^cells, CD43 signals did not affect the transcription of either the IFNγ or IL-4 genes (panels C and D) yet those of the TcR induced IFNγ transcription with no change in IL-4 mRNA levels. The joint signals from CD43 and the TcR stimulated IL-4 transcription over the levels found in response to TcR signals, consistent with the fact that in CD8^+ ^cells, the combination of CD43- and TCR-dependent signals did not result in a higher proportion of INFγ^+ ^cells (Figure 1). CD43 expression was similar in all the populations of cells, as evaluated by L10 staining (data not shown).

### Effect of CD43 signals in the transcription of the T-bet and GATA-3 genes

**Figure 3:**
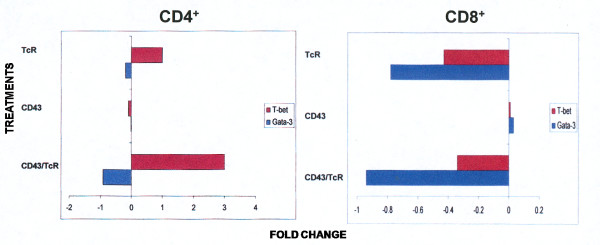
**CD43 and TcR signals induce the transcription of the T-bet gene in CD4^+ ^T cells and inhibit GATA-3 gene transcription in both CD4^+ ^and CD8^+ ^T cells**. T cells were treated for 6 h, as indicated in Figure 1. Total RNA was extracted from CD4^+ ^(panel A) or CD8^+ ^T cells (panel B) and subjected to real time RT-PCR analysis for T-bet or GATA-3 mRNAs as compared to the internal control GAPDH. The graphics show the fold change in the levels of the messengers, as compared to untreated cells (base line). A representative of four independent experiments is shown. Each point is the mean of duplicates which differed by <2%.

Differentiation into effector T cells is accompanied by the selective expression of lineage-specific transcription factors. T-bet and GATA-3 have been described as the signature transcription factors for differentiation of T1 and T2 cells, respectively [22,23]. Although CD43 signals alone had no effect on the transcription of T-bet or GATA-3 genes, in cooperation with those of the TcR, led to a marked inhibition of the GATA-3 gene transcription in both, CD4^+ ^or CD8^+ ^T cells (Figure 3A and 3B). Moreover, in CD4^+ ^T cells, the combined signals of CD43 and the TcR induced the transcription of the T-bet gene (Figure 3B). The CD43-mediated increase in T-bet and inhibition of GATA-3 genes transcription, could direct CD4^+ ^T cell differentiation into the T1 pattern.

### CD43 ligation increased the expression of IL-12 and IFNγ receptor

**Figure 4:**
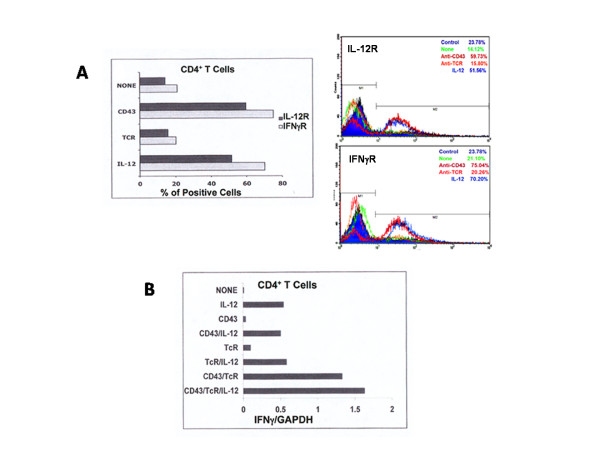
**CD43 induces IFNγR and IL-12R expression and cooperates with IL-12 for IFNγ transcription in CD4^+ ^T cells**. CD4^+ ^T cells were left non-stimulated (none), or were stimulated for 24 h (A), by antibody ligation of CD3 (TcR) or CD43 followed by cross-linking with a secondary antibody or were stimulated for 12 h (B) by antibody ligation of CD3 (TcR), CD43, or the simultaneous ligation of both molecules, in the presence or absence of IL-12. A) Cells were collected and stained with FITC-labelled anti-IL-12R and PE-labelled anti-IFNγR antibodies and analyzed by flow cytometry. The graphics show the fold change in the expression levels of the receptors, as compared to untreated cells. The corresponding histograms are shown to the left of the graph. B) Total RNA was extracted and subjected to real time RT-PCR analysis for IFNγ mRNA contents, as compared to the internal control GAPDH. A representative of two (A) or three (B) experiments is shown.

Cytokine expression depends on signals from co-receptors as well as from cytokines. Upon interaction with their receptors, IL-12 and IFNγ induce T1- and IL-4 T2-differentiation patterns [2,9]. We investigated the effects of signals from the TcR, CD43 and IL-12 in the membrane-expression levels of IL-12 (IL-12R) and IFNγ (IFNγR) receptors in CD4^+ ^T cells. IL-12R and IFNγR are expressed at low levels in non-stimulated CD4^+ ^T cells [24,25]. CD43-ligation, but not that of the TCR, induced an increase in the percentage of IL-12R^+ ^or IFNγR^+ ^cells. As expected, the IL-12 signals augmented the percentage of IL-12R^+ ^and IFNγR^+ ^cells (Figure 4).

### IL-12 signals augment the induction of IFNγ in response to CD43 and TcR signals in CD4^+ ^T cells

We next investigated if CD43 signals affected the IL-12-driven transcription of IFNγ in CD4^+ ^T cells. The individual signals from CD43 or the TcR did not alter the IL-12-induced IFNγ transcription. The combination of CD43, the TcR and IL-12 signals led, however, to a 20% increase of the IFNγ gene transcription over that resulting of the joint signals of CD43 and the TcR (Figure 4B). This enhancement was also reflected at the protein level, because the percentage of IFNγ^+ ^cells increased when IL-12 was added together with anti-TCR and anti-CD43 mAbs (data not shown). As expected, IL-12 signals induced IFNγ transcription. These results suggest that CD43-mediated signals favour the IL-12-driven differentiation into T1 cells.

### CD43 signals induce the co-clustering of IFNγR and the TcR

**Figure 5:**
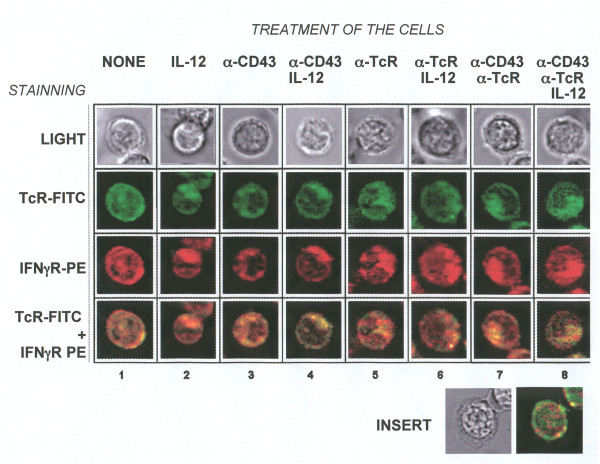
**CD43, the TcR and/or IL-12 signals induce the redistribution of the TcR and IFNγR**. CD4^+ ^T cells were treated for 12 h as shown and stained with FITC-labelled anti-CD3 and PE-labelled anti-IFNγR before analysis by fluorescence microscopy. Insert shows a non-stimulated CD8^+ ^T cell.

The membrane distribution of IFNγR and the TcR has been shown to determine the effector phenotype of lymphocytes. When IL-12 is present during activation, the co-capping of the TcR and IFNγR gives rise to a T1 differentiation pattern [26]. We found that in CD4^+ ^T cells, the TcR and IFNγR always co-localized, whether the cells were stimulated or not (Figure 5). The separate signals of CD43, the TCR and IL-12 all induced a significant increase in the percentage of cells with TcR and IFNγR co-clustering (Table 1). The joint signals of CD43 and the TcR increased this percentage over that resulting of the individual stimuli. Addition of IL-12 simultaneous with CD43 and TCR ligation did not augment the proportion of cells with co-clustered receptors over that found for CD43- or CD43- and TcR-treated cells. The addition of IL-12 to TcR-ligated cells, however, augmented the proportion of cells with co-clustered receptors as related to TcR-ligated cells (Table 1).

**Table 1:** Co-clustering of TcR and IFNγR in response to CD43, the TcR and/or IL-12 or IL-4 signals

**TREATMENT**	**% OF CELLS WITH CO-CLUSTERED RECEPTORS (Mean ± SD)**
NONE	30.6 ± 4.7
CD43	59.3 ± 4.5
IL-12	53.6 ± 4.5
CD43/IL-12	54.3 ± 1.5
IL-4	38.3 ± 5.5
CD43/IL-4	39.3 ± 3.6
TCR	54.3 ± 2.3
TCR/IL-12	60.6 ± 2.0
TCR/IL-4	29.3 ± 3.6
CD43/TCR	67.3 ± 1.5
CD43/TCR/IL-12	64.6 ± 4.2
CD43/TCR/IL-4	28.7 ± 3.0

CD4^+ ^T cells were left non-stimulated or were stimulated for 12 h by antibody ligation of the TcR, CD43, or the simultaneous ligation of both molecules, in the presence or absence of IL-12 or IL-4. Cells were stained with FITC-labelled anti-CD3 and PE-labelled anti-IFNγR antibodies and analyzed with a fluorescent microscope. For each treatment we counted 100 cells and determined the percentage of cells with co-clustered receptors over those with an even distribution of receptors. Statistical significance was evaluated by the Student T test. Differences were significant with p < 0.01 for CD43, the TcR or IL-12 signals as compared with non-stimulated cells and p < 0.05 for the joint signals of CD43 and the TcR as compared with anti-CD3 or anti-CD43. The addition of IL-12 to the antibody treated cells resulted in a significant difference only for the anti-TCR treatment (p < 0.05). IL-4 significantly inhibited the co-clustering of the IFNγR and the TcR in all the antibodies treatments (p < 0.01).

The localization and topography of the TcR and IFNγR clusters also changed in response to the different stimuli. In IL-12-treated cells, both receptors redistributed towards one half of the cell in a few big clusters (Figure 5, lane 2). CD43 signalling resulted in smaller clusters all over the cells or towards one pole (lane 3), whereas the joint signals from CD43 and IL-12 (lane 4) or of CD43 and the TcR (lane 7) promoted the formation of a co-cap of the TcR and IFNγR in one extreme of the cells. No further effect was observed as a result of the combined signals of CD43, the TcR and IL-12 (lane 8). Signals from the TcR alone induced the redistribution of the TCR and IFNγR towards one half of the cell, and the addition of IL-12 did not change the phenotype (lanes 5 and 6). In CD8^+ ^T cells, the TcR and IFNγR did not co-localize and CD43 signals did not affect the aggregation of either receptor (Figure 5 insert)

Finally, we examined whether IL-4 signals disrupted the TcR and IFNγR co-clustering. In murine cells, It has been shown that IL-4 signals inhibit the co-clustering of the TcR and IFNγR and that, as a consequence, cells differentiate into T2 [26]. In agreement with this, IL-4 signals inhibited the redistribution of receptors resulting from IL-12, CD43 or TcR engagement alone or in different combinations (Table 1).

## Discussion

We evaluated the effect of CD43 signals alone or in combination with those of the TcR on IFNγ expression in freshly isolated CD4^+ ^or CD8^+ ^T cells and in T1 or T2 effector cells. In freshly isolated CD4^+ ^T cells, CD43 signals alone led to a small increase in the percentage of IFNγ^+ ^cells, and synergized with TCR signals in the induction of IFNγ^+ ^cells. This was not observed in CD8^+ ^T cells stimulated under the same experimental conditions. On effector cells, CD43 signals alone lead to a strong induction of IFNγ not only in T1 but also in T2 CD4^+ ^cells and to a lower extent in CD8^+ ^cells. These data suggest a specific role of CD43 signals in IFNγ expression.

The biology, function and distribution of CD4^+ ^and CD8^+ ^T cells during an immune response are different. We found that CD8^+ ^T cells are less responsive to CD43-dependent signals. A differential response of CD4^+ ^and CD8^+ ^T cells to co-receptors has also been observed for the TNF family co-receptors, 4-1BB and OX40. 4-1BB preferentially activates CD8^+ ^cells, and inhibits of CD4^+ ^T cells. Conversely, OX40 is mainly stimulatory for CD4^+ ^T cells [27,28]. The differences encountered between CD4^+ ^and CD8^+ ^T cells to CD43 signals are more significant in the primary activation signals. This is consistent with data reporting that CD8^+ ^cells are less dependent on co-receptors for activation [29].

Lineage-commitment is characterized by the cytokine profiles that cells populations secrete and also by the presence of lineage-specific transcription factors that mediate epigenetic changes, favouring the expression of those cytokines [30]. In addition to the IL-12- and the IFNγ-induced transcription factors STAT-4 and STAT-1, T1 cells express T-bet, which is considered to be the T1 signature transcription factor [22]. In contrast, IL-4-induced STAT-6 as well as GATA-3 are characteristic of T2 cells. [23]. Here we report that the joint signals of CD43 and the TcR inhibited GATA-3 transcription, both in CD4^+ ^and CD8^+ ^T cells, and that those signals increased the transcription of T-bet in the CD4^+ ^population only. To be able to respond to T1 stimulatory cytokines, T cells up-regulate the expression of the high affinity chains of the IL-12R and IFNγR. With specific antibodies for the α chain of IFNγR or the β1 chains of IL-12R, we evidenced that CD43 signals induce the membrane expression of both receptors, thereby contributing to the sensitivity of the cells towards T1-differentiating cytokines.

The distribution of the TcR relative to that of IFNγR has also been shown to be determinant for T cell commitment, suggesting a crosstalk between the TcR and IFNγ signalosomes [26]. We observed that in CD4^+ ^T cells, CD43 signals either alone or in combination with TcR and/or IL-12R ligation led to the co-clustering of IFNγR with the TcR, further adding to the T1-differentiating effects of CD43. In contrast, in CD8^+ ^cells, CD43 signals did not affect the membrane distribution of IFNγR and the TcR in the membrane. Moreover, we did not find that these receptors co-localized in CD8^+ ^T cells, where it has been reported that the redistribution of molecules during the immunological synapses is strongly dependent on CD8 engagement [31].

## Conclusion

All together our results suggest that CD43 signals favour CD4^+ ^T cell differentiation to a T1 pattern. In conjunction with TCR-dependent signals, CD43 induced T bet and IFNγ genes and inhibited those of GATA-3 and IL-4. Concomitantly, CD43-treated cells became more sensitive to the T1-inducing cytokines IFNγ and IL-12 through the up-regulation of their receptors. Part of this could be achieved by the CD43-mediated co-clustering of the TcR and IFNγR.

In CD8^+ ^T1 or T2 effector cells, CD43 signals moderately induced IFNγ expression. In freshly isolated CD8 cells, however, CD43 signals induced IL-4 transcription, and failed to induce IFNγ expression. In CD4^+ ^cells, the TcR and IFNγR co-localize and cluster together in response to CD43, the TCR or IL-12, whereas in the CD8^+ ^T cells, these receptors did not co-localize and did not re-distribute in response to the same stimuli. Resolution of whether the differential responses of the CD4^+ ^and CD8^+ ^populations to CD43-specific signals could have a therapeutic use to dissociate T cell-mediated cytotoxicity of the inflammatory response requires further experiments.

## Methods

### Reagents

For ligation of CD43, we used the monoclonal antibody L10 from Caltag laboratories (Burlingame, CA). Anti-CD3 mAb (clone SPV-T3b) and rabbit anti-mouse IgG were from Zymed (San Francisco, CA). Antibodies for flow cytometry and immunocytochemistry were from BD PharMingen (San Diego, CA); using clone 4S. B3 for staining IFNγ, clone 2.4E6 for staining the β1 chain of IL-12R and clone GIR-208 for the α chain of IFNγR. Ficoll-Hypaque was from Sigma. dNTPs were from Roche Molecular Biochemicals (Indianapolis, IN). Murine mammary tumour virus reverse transcriptase, RNase inhibitor and cytokines were from Invitrogen (Carlsbad. CA). All reagents for real-time PCR were from Applied Biosystems (Foster City, CA).

### Cell preparations

Leukocyte concentrates from healthy adult donors were provided by the Blood Bank from Hospital de Zona of the Instituto Mexicano del Seguro Social in Cuernavaca. CD4^+ ^or CD8^+ ^T cells were isolated by RossetteSep separation, following the manufacturer s instructions (StemCell Technologies, Vancouver). Cells were cultured as previously described [19]. The purity of cell populations, assessed by flow cytometry, was over 95% in all cell preparations. The remaining cells being 0.5–3% the opposite T cell subset and less than 1% CD19^+ ^cells. Before experiments, T cells were arrested for 12 h in RPMI supplemented with 2% foetal calf serum. Subsequent manipulations were done in this medium. T1 or T2 cells were differentiated for one week as described [21]. For cell activation, T cells (5 × 10^6 ^cells/ml) were incubated in 24-well plates with the following antibodies, alone or in combination: L10, 1 μg/ml; anti-CD3, 1 μg/ml, cross-linked with rabbit anti-mouse IgG (1 μg/ml). Addition of the secondary antibody alone or in the presence of irrelevant antibodies did not elicit cell responses (data not shown). In some experiments, cells were incubated in the presence of IL-12 (10 ng/ml) or IL-4 (10 ng/ml).

### Real-time PCR

Total RNA was obtained using TRizol (Invitrogen, Inc.), following the manufacturer s instructions. cDNA was synthesized from 1 μg of total RNA by standard reverse transcription conditions, using primer (dT), in a final volume of 30 μl. cDNA was diluted 1/10 and 2 μl of the diluted sample were used for amplification in a 5700 Gene Amp equipment (Applied Biosystems) with 15 min denaturation and 1 min annealing/extension cycles. For cytokine gene amplification we used the PDAR designed reagents for Taqman amplification from Applied Biotechnology, with the PDAR probe for GAPDH as comparison. The amplification of T-bet, GATA-3, and GAPDH, used again as control gene, was done with SYBR green labelling, using the following oligonucleotides: GATA-3: 5' CCC AAG AAC AGC TCG TTT AAC C, 3' AGA TGT GGC TCA GGG AGG ACA T; T-bet: 5' CCA CCT GTT GTG GTC CAA GTT, 3' TCC CTG CTT GGT GAT GAT CAT; GAPDH: 5' ACC TGA CCT GCC GTC TAG AAA, 3' CCTGCT TCA CCA CCT TCT TGA T. These oligonucleotides were designed with primer express software (Applied Biosystems). All the primers have equivalent melting temperatures and give amplicons of 100–120 base pairs.

### Staining for Flow Cytometry

T cells were stained for flow cytometry (FACS) analysis as described [19]. Cells were analyzed with a FACScallibur with the CELLQUEST program (Becton and Dickinson, San José, CA). For cytokine staining, cells were treated with or without the different stimuli for 12 h in the presence of brefeldin, as indicated by the manufacturer (BD). Intracellular cytokine staining was performed with kits from BD.

### Immunocytochemistry

Cells were incubated in 24-well culture dishes, on 10% poly-L-lysine pre-treated cover-slips, in the presence or absence of any of the following stimuli alone and in different combinations: anti-CD43 mAb, anti-CD3 mAb, IL-12 or IL-4. After 12 h incubation, the cover-slips were collected and the adhering cells were fixed with 100% methanol and blocked with 5% serum albumin in PBS. Cells were then stained with anti-IFNγR labelled with phycoerythrin and anti-CD3 labelled with FITC. Controls were isotype matching irrelevant antibodies labelled with the same dyes. Cover-slips were soaked in 10% glycine in PBS and attached to microscope slides for microscopic evaluations with a Zeiss Optical slicing fluorescent microscope Axiovert 200 M (Carl Zeiss, Oberkochen). We used an amplification of 63× and performed 40 optical cuts of 0.2 μm. The cells were exposed to illumination for 6–8 s. Images were captured with an Axiocam MR camera and analyzed with the Axiovision Rel 4.4 software. The integration of the cuts is shown.

## Authors' contributions

All authors read and approved the final manuscript

ORP contributed with the experiments of real time PCR, the cell preparations and some of the repetitions of intracellular cytokine analysis. He helped to draft the manuscript.

DLEZ contributed with the microscopy data and its statistical analysis.

GMRM contributed with the experiments and analysis of IFNγR and IL-12R expression.

MGCB contributed with the experiments and analysis of intracellular cytokine expression.

FREG contributed with the design of experiments of analysis of cytokine expression and directed MCB's work.

GRS contributed with the training of DLEZ in fluorescence microscopy and the design and analysis of microscopy experiments.

YR contributed with the conception of the study and participated in the analysis and interpretation of data and in the preparation of the manuscript.

MAS conceived the study, got the funding of the work, directed the work of ORP, DLEZ and GMRM, and drafted the manuscript.
